# The Clinical Challenge of Sepsis Identification and Monitoring

**DOI:** 10.1371/journal.pmed.1002022

**Published:** 2016-05-17

**Authors:** Jean-Louis Vincent

**Affiliations:** Department of Intensive Care, Erasme Hospital, Université libre de Bruxelles, Brussels, Belgium

## Abstract

Jean-Louis Vincent outlines why combinations of biomarkers will be central to the future of sepsis diagnosis.

**Summary Points**

- Early treatment of sepsis is associated with improved outcomes so that rapid diagnosis is important.
- The diagnosis of sepsis in critically ill patients is challenging, because it can be complicated by the presence of inflammation as a result of other underlying disease processes and prior use of antibiotics making cultures negative.
- Culture-dependent diagnosis of infection is slow, and biomarkers may provide a more rapid means of ruling in or out infection.
- Given the complexities of the sepsis response, no one biomarker will be sufficient to diagnose sepsis. Combinations of biomarkers are needed, and new technology is helping to speed the development of such panels.
- However, such tools cannot be used alone, and they must be seen as complementary to a careful clinical assessment and other laboratory signs.

## Introduction—The Clinical Problem

Infections are common in people of all ages and around the globe. In most individuals, the host response is adequate to deal with the potential threat, and little treatment is needed other than a short course of appropriate antibiotics if the origin is bacterial. However, in some cases, infection can be associated with an inadequate or inappropriate host response, and when this results in the development of organ dysfunction, the term “sepsis” is used [1–3]. Sepsis can be associated with viral or fungal infections, but the inflammatory response is generally less marked in these cases, and the majority of patients with sepsis will have a bacterial infectious source. Such patients are critically ill and likely to rapidly deteriorate into septic shock and multiple organ failure if not treated quickly and effectively. Indeed, sepsis is associated with mortality rates of around 30%, although these vary according to geographical location [4]. Most of the available epidemiological data on sepsis come from developed countries, and few data are available describing sepsis patterns and outcomes in poorer income and lower resource countries [5].

There is no specific treatment for patients with sepsis, and management therefore relies on infection control—with source removal and effective antibiotics—and organ function support [6]. There is good evidence that early treatment is associated with improved outcomes in these patients [6–8], and the ability to recognize the condition as soon as possible is therefore important, so that treatment can be started early in the course of disease to prevent deterioration [6]. However, the early diagnosis of patients with sepsis remains a challenge for clinicians at the bedside.

## “Diagnosis” of Infection and Sepsis

Infection is defined as “a pathologic process caused by the invasion of normally sterile tissue or fluid or body cavity by pathogenic or potentially pathogenic microorganisms” [9]. Sepsis is defined as the presence of organ dysfunction occurring as the result of a dysregulated host response to an infection [1–3]. In a young patient with an obvious meningococcal rash, high fever, and altered mental status, diagnosis of sepsis is fairly straightforward, but this is not always the case, especially amongst the critically ill population with multiple comorbidities and other ongoing disease processes.

**Fig 1:**
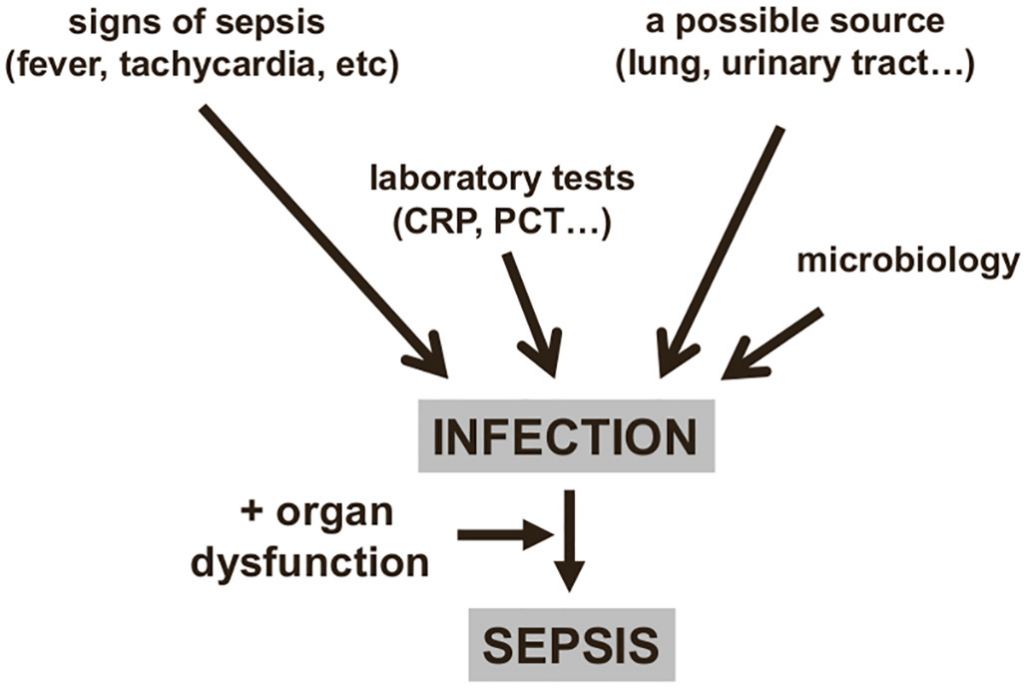
Diagnosing sepsis from infection. An infection can be identified from clinical signs and microbiology findings, providing a diagnosis of sepsis if organ dysfunction is also present. CRP: C-reactive protein; PCT: procalcitonin.

Infection is typically identified by three types of information (Fig 1):

- The onset of clinical signs and symptoms of a host response: Fever and chills are the most typical clinical reaction. The biological response is characterized by an increase (sometimes a decrease in severe cases) in both the white blood cell count and in concentrations of inflammatory markers, e.g., blood C-reactive protein (CRP) or procalcitonin (PCT).
- The presence of signs of infection: for example, dysuria and smelly urine; respiratory symptoms with abnormal chest auscultation and typical radiographic chest infiltrates; purulent wounds; signs of meningitis.
- Proven microbiological invasion of a sterile environment: a positive peritoneal tap in a cirrhotic patient or signs of superinfection in a nonsterile environment (gastroenteritis) are good examples.

However, not all these elements are always present: for example, an immunosuppressed patient may not develop fever, and a source of infection is sometimes impossible to identify. Moreover, while many critically ill patients have some degree of hyperthermia, this does not necessarily imply the presence of an infection [10,11]; also, some patients, particularly the elderly, may have hypothermia, and this is associated with worse outcomes [12]. Similarly, tachycardia and tachypnea are common in critically ill patients, and the white blood cell count, even with differential, is of little interest in the acutely ill, as changes may be due to any form of stress. Moreover, leukopenia can be observed in severe cases when leukocytes are activated in mass and trapped in the periphery. For these reasons, the systemic inflammatory response syndrome (SIRS) criteria are inadequate to diagnose sepsis [13] and should no longer be used [1].

**Fig 2:**
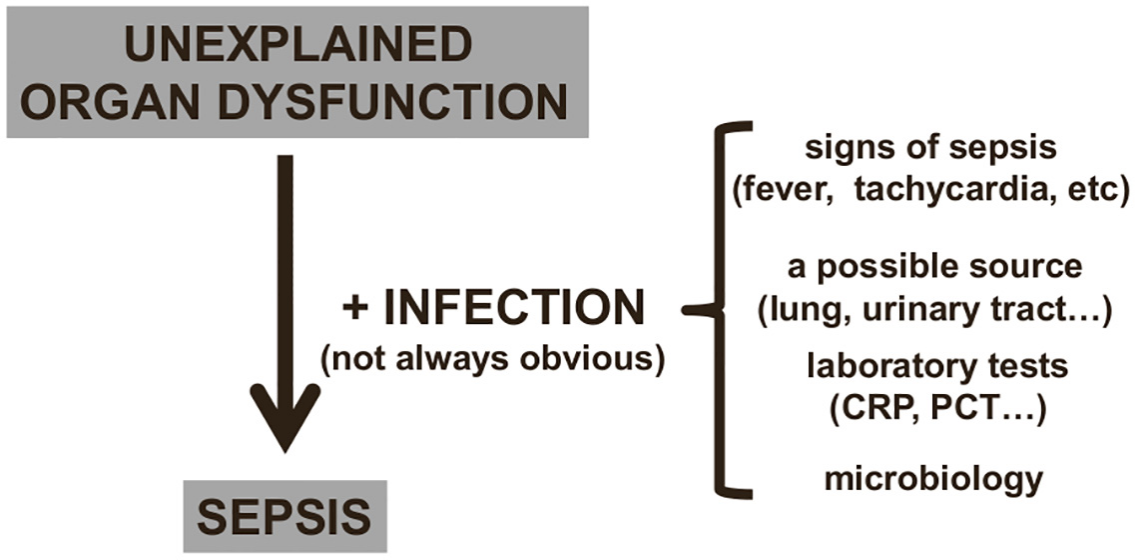
Diagnosing sepsis from organ dysfunction. In critically ill patients, infection can be difficult to identify (see text), and sepsis is often suspected by the presence of unexplained organ dysfunction, which should lead to a search for the underlying infection. CRP: C-reactive protein; PCT: procalcitonin.

Many critically ill patients will also recently have received or be receiving antimicrobial therapy, which can render microbial cultures negative. In a multicenter one-day point prevalence study, 71% of the 13,796 adult intensive care unit (ICU) patients included were receiving antibiotics on the day of the survey [14]. In the same study, 30% of cultures from infected patients were negative [14]; other studies have similarly reported that 30%–40% of ICU patients with sepsis have negative cultures [15,16]. In such situations, an otherwise unexplained organ dysfunction may be the only clue to a possible diagnosis of sepsis (Fig 2). Even when cultures are positive, several days may be needed before results are available. New culture-independent techniques for pathogen detection and identification, including polymerase chain reaction, will help speed initial microbial identification in the near future [17–19].

Given these difficulties with diagnosis, it is not surprising that sepsis can sometimes be under- or overdiagnosed, perhaps especially by health care staff who less frequently encounter such patients. A typical example of underdiagnosis would be a patient who is becoming confused and/or hypoxemic and/or has a low platelet count but has no clear clinical evidence of an infection so that sepsis is not even considered. A typical example of overdiagnosis would be a postoperative patient with fever who is given antibiotics although fever is a reaction to the surgical trauma rather than to an infection. In one telephone survey of more than 1,000 hospital physicians in Europe and the United States, half of whom were intensive care specialists, 86% stated that that the symptoms of sepsis could easily be misattributed to other conditions, and 45% felt that they sometimes missed a diagnosis of sepsis [20]. It is difficult to establish an objective picture of how many cases of sepsis are over- or underdiagnosed because reported rates of sepsis currently rely on different definitions [21] or on different hospital disease identification or billing codes [22].

## The Role of Sepsis Markers

Considering the mentioned difficulties in diagnosing sepsis, the availability of accurate sepsis (bio)markers to facilitate diagnosis could be of use to enable timely appropriate treatment to be started, thus optimizing a patient’s chances of survival. More than 170 biomarkers have been proposed and assessed clinically [23], including various cytokines, cell surface markers, receptors, complement factors, coagulation factors, acute phase reactants, and many others [24–26], but none has 100% specificity for sepsis. Perhaps the most widely studied biomarker of sepsis is CRP, whose role in host defense against bacteria has been known about for almost 100 years [27]. However, CRP is sensitive but not very specific, being increased in all inflammatory disorders, including after uncomplicated surgery [28]. PCT, first proposed as a biomarker in 1993 [29], is perhaps a more specific marker than CRP [30–34], although it is also increased in other inflammatory conditions, such as pancreatitis or after polytrauma or major surgery [35–37].

Sepsis markers can be helpful in answering three types of general questions (Table 1).

- The first question relates to their role in helping to identify—or perhaps more importantly rule out—an infection. Infection is not an all-or-none phenomenon, and there are "gray areas" where one can never really be certain that an infection was present or absent. Because of their high sensitivity, sepsis markers are usually more helpful at ruling out than at ruling in an infection. This is particularly true in critically ill patients, who often have some inflammatory response, but do not always have infection or require antibiotic administration. Hence, sepsis markers, by ruling out infection, could help decrease the use of unnecessary antibiotics, limit the use of excessive imaging procedures in search of a possible source, and encourage the clinician to search for alternative diagnoses. One example of this use for biomarkers was demonstrated by Christ-Crain and colleagues using PCT to rule out infections in febrile patients presenting to an emergency department [38]. In patients with suspected lower respiratory tract infection, use of antibiotics was more or less discouraged (<0.1 mcg/L or <0.25 mcg/L) or encouraged (≥0.5 mcg/L or ≥0.25 mcg/L) based on the PCT concentration. The result was a significant reduction in antibiotic use [38].
- The second question relates to their role in assessing the severity of disease, primarily for triaging decisions. For example, whether or not to admit a patient from the emergency room or general ward to the ICU. An example of this use for biomarkers was demonstrated by Giamarellos-Bourboulis et al. [39] in 1,156 hospitalized patients, showing that mortality rates were 2.6 times higher in patients presenting with sepsis on the general ward with a PCT concentration >0.12 ng/mL than in those with lower PCT levels. The authors suggested that PCT concentrations could thus be used to help identify which patients may benefit from ICU admission [39].
- The third question relates to their role in monitoring a patient's response to therapy. For this role in particular, trends in concentrations over time are clearly of more value than single measurements. Again, PCT and CRP are the most widely studied biomarkers in this context. Karlsson et al. [40] reported that mortality rates were substantially lower in septic patients in whom the PCT concentration decreased by more than 50% over 72 hours than in the other patients (12% versus 30%, *p* = 0.007). Similarly, the pattern of change in CRP concentrations correlated with the individual clinical course in patients with community-acquired sepsis [41]. A progressive decline in CRP or PCT concentrations can be used to guide earlier discontinuation of antibiotic therapy, without major risks [42]. But an increase in CRP concentrations in the first 48 hours of therapy suggests that antibiotic therapy may be ineffective and need reevaluation [43]. PCT levels have been used to guide antibiotic therapy in several clinical trials in different groups of infected patients with promising results on antibiotic use [44,45]. However, using PCT concentrations in an antibiotic-escalation strategy is not a wise strategy, as it may result in worse outcomes [46]. Clearly, clinical decisions should not be based just on the concentrations of a single biomarker but must include evaluation of the clinical status of the patient and other hemodynamic and laboratory parameters.

**Table 1:** How a biomarker, levels of which increase in sepsis, can be used to answer clinically important questions.

Clinical question	Sepsis Marker	
	Increases or stays high	Low or decreasing
Is the patient infected?	Suggests antibiotics should be considered	Suggests may not need antibiotics
	Suggests need to search for a source of infection	Suggests additional investigations for infection could be postponed
Is the condition serious?	Suggests admission to hospital or intensive care unit may be warranted	Suggests patient discharge home or transfer to regular floor could be considered
Is the patient responding to treatment?	Suggests antibiotic therapy may need to be reassessed	Suggests treatment is effective and could be continued (reassurance)
	Suggests may need to assess need for (re)operation	Suggest antibiotic therapy could perhaps be stopped?

## The Search for a Perfect Sepsis Marker?

**Fig 3:**
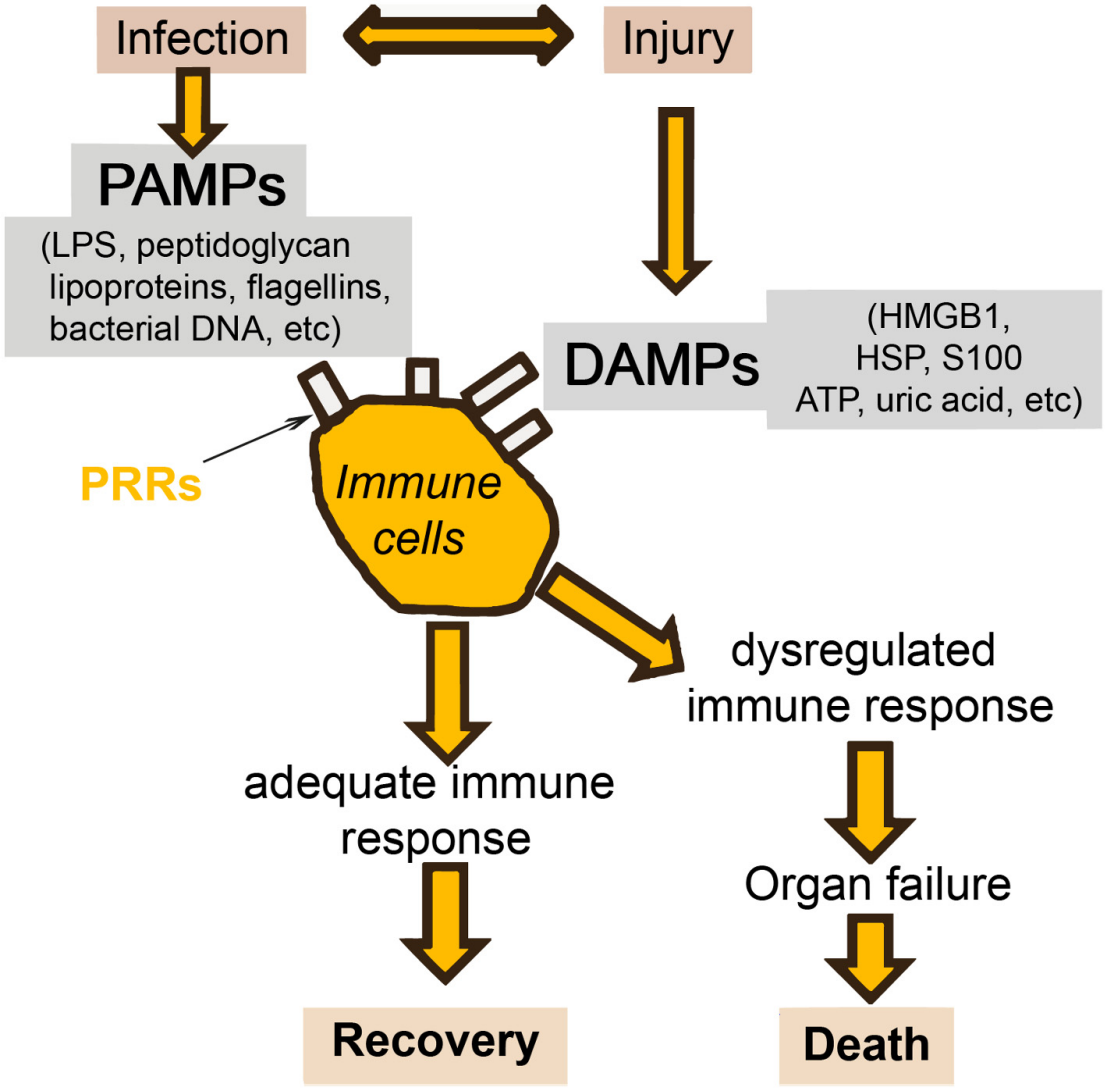
Simplification of the host response to an infection. Injury (via DAMPs) and infection (via PAMPs) can stimulate the same inflammatory reaction via the PRRs. HMGB: high mobility group box protein; HSP: heat shock protein.

There will always be a gray area between definitive sepsis and definitive absence of sepsis. This is due to two important factors. The first is the symbiosis of microorganisms in our gut. It is likely that altered tissue perfusion is associated with gut hypoperfusion, with translocation of microorganisms that can in turn activate the immune system. The potential contribution of gut translocation to sepsis and organ failure was proposed a number of years ago, with the famous phrase "the gut is a motor of multiple organ failure" [47]; although difficult to prove, this concept still makes sense. The second factor is that a similar inflammatory response can also arise from noninfectious events, so that the phenotype of a patient with sepsis can be clinically identical to that of a patient with, for example, trauma, pancreatitis, or burns [1]. It is well known that the sepsis response can be triggered by evolutionarily-conserved pathogen-produced substances, such as endotoxin, called pathogen-associated molecular patterns (PAMPs). But damage-associated molecular patterns (DAMPs) released by damaged host cells, for example in trauma or burns injury, can induce the same inflammatory reaction through the same receptors (pattern recognition receptors, PRRs) (Fig 3) [48,49].

Given these complexities, and the different times at which individual biomarker levels are elevated, I do not believe that a single marker will ever be of use alone to diagnose sepsis in the heterogeneous critically ill patient population. Rather, research is increasingly focusing on the development of combinations or panels of biomarkers, potentially combined with clinical signs. In patients with SIRS suspected of having community-acquired infections, Kofoed et al. found that a combination of six biomarkers (soluble urokinase-type plasminogen activator, soluble triggering receptor expressed on myeloid cells [sTREM]-1, macrophage migration inhibitory factor, CRP, PCT, and neutrophil count), had a significantly greater area under the curve (AUC) for a bacterial cause of inflammation than did any of the individual markers [50]. Similarly, Gibot et al. reported that a combined score of PCT, sTREM-1, and the polymorphonuclear CD64 index diagnosed sepsis better than any of the individual biomarkers [51].

Use of new genomic, proteomic, and metabolomic technology applied to large datasets is changing the ways in which effective biomarkers are identified and validated [52–56]. For example, Pena et al. analyzed microarrays from 593 patients in 11 cohorts to develop a gene endotoxin tolerance signature which was strongly associated with the development of sepsis and severity of disease [54]. Langley et al. [56] analyzed metabolomic and transcriptomic datasets from primates with *Escherichia coli* sepsis and identified a four-metabolite panel that diagnosed sepsis in two human cohorts with AUCs of 0.78 and 0.82, respectively. Most recently, McHugh et al. [57], using microarray analysis in a development cohort, identified a molecular classifier of four RNA biomarkers, and showed in validation cohorts that it was able to distinguish patients with sepsis from those without with an AUC of 0.88 and was better at discriminating sepsis from infection-negative systemic inflammation than were clinical and laboratory parameters, including PCT.

This is a rapidly advancing and exciting field. Clearly, further studies are needed to determine which combinations of which markers will give the best performance in which patients. The industry needs to be encouraged to collaborate with researchers and invest more in this field so that reliable biomarkers can be developed and commercialized for the benefit of our patients. Importantly, a marker or a combination of markers will never replace a careful clinical assessment and must be considered as a complementary tool to diagnosis.

## Conclusions and Perspective

Diagnosing sepsis early is crucial so that appropriate treatment can be started promptly and patients given the best possible chances of survival. The lack of progress in sepsis diagnosis using biomarkers is partly related to the fact that we have concentrated on changes in single markers, whereas the sepsis response involves multiple players present at different times during the disease process. We are never going to find a single ideal marker for sepsis. The future lies with more research focused on the use of panels or combinations of markers. Nevertheless, currently available sepsis markers can already assist in identifying and even more importantly ruling out sepsis, assessing disease severity, and indicating the need to re-evaluate ongoing therapy, when used in combination with repeated clinical evaluation. Use of biomarkers in the future will help improve patient outcomes by improving diagnostic accuracy, reducing the time to effective treatment, and limiting unnecessary tests and treatments. On a more global level, the resultant improved antibiotic stewardship should help optimize antibiotic use, thus increasing patient safety, reducing costs and reducing the development of antibiotic resistance.
